# Population Dynamics and Range Expansion in Nine-Banded Armadillos

**DOI:** 10.1371/journal.pone.0068311

**Published:** 2013-07-03

**Authors:** William J. Loughry, Carolina Perez-Heydrich, Colleen M. McDonough, Madan K. Oli

**Affiliations:** 1 Department of Biology, Valdosta State University, Valdosta, Georgia, United States of America; 2 Carolina Population Center, University of North Carolina, Chapel Hill, North Carolina, United States of America; 3 Department of Wildlife Ecology and Conservation, University of Florida, Gainesville, Florida, United States of America; Texas Tech University, United States of America

## Abstract

Understanding why certain species can successfully colonize new areas while others do not is a central question in ecology. The nine-banded armadillo (*Dasypus novemcinctus*) is a conspicuous example of a successful invader, having colonized much of the southern United States in the last 200 years. We used 15 years (1992–2006) of capture-mark-recapture data from a population of armadillos in northern Florida in order to estimate, and examine relationships among, various demographic parameters that may have contributed to this ongoing range expansion. Modeling across a range of values for γ, the probability of juveniles surviving in the population until first capture, we found that population growth rates varied from 0.80 for γ = 0.1, to 1.03 for γ = 1.0. Growth rates approached 1.0 only when γ ≥0.80, a situation that might not occur commonly because of the high rate of disappearance of juveniles. Net reproductive rate increased linearly with γ, but life expectancy (estimated at 3 years) was independent of γ. We also found that growth rates were lower during a 3-year period of hardwood removal that removed preferred habitat than in the years preceding or following. Life-table response experiment (LTRE) analysis indicated the decrease in growth rate during logging was primarily due to changes in survival rates of adults. Likewise, elasticity analyses of both deterministic and stochastic population growth rates revealed that survival parameters were more influential on population growth than were those related to reproduction. Collectively, our results are consistent with recent theories regarding biological invasions which posit that populations no longer at the leading edge of range expansion do not exhibit strong positive growth rates, and that high reproductive output is less critical in predicting the likelihood of successful invasion than are life-history strategies that emphasize allocation of resources to future, as opposed to current, reproduction.

## Introduction

Understanding why some species are able to successfully colonize new areas while others do not is a key question in ecology and conservation biology [1], [2]. A number of critical features of successful invaders have been proposed; among these are possession of certain life-history characteristics [3], [4], ecological release from former predators and/or pathogens [5], and various anatomical and behavioral features that may increase adaptability to novel environments [6].

In addition to the aforementioned, intrinsic features of animal populations must inevitably play some role in determining the success of any invasion. For example, for a range to expand it is only logical to assume that populations produce sufficient individuals such that some leave current areas to colonize new ones. This could be accomplished by high reproductive output, high survivorship, or some combination of the two. Consequently, models to estimate population growth rates, coupled with prospective and retrospective perturbation analyses to identify parameters that most influence these rates, can provide valuable insights into the factors that might promote range expansion in a particular species.

Among mammals, the nine-banded armadillo (*Dasypus novemcinctus*; hereafter referred to as “armadillo”) is a dramatic example of a successful invader. Although widely distributed across much of the Americas [7], armadillos have colonized the United States only recently. First recorded in the Rio Grande valley of Texas in the 1840 s [8], the species has subsequently expanded its range quite rapidly, so that it is now found from eastern New Mexico [9] to South Carolina [10], and as far north as Nebraska [11], southern Illinois and Indiana [12]-[14], and the Cumberland Plateau of Tennessee [15]. No quantitative assessments have been conducted but speculation about factors promoting this extensive range expansion have focused on the seemingly high tolerance of armadillos to human disturbance, which underscores their flexibility in adapting to a wide range of environmental conditions, and the occurrence of polyembryony, whereby females produce litters of genetically identical quadruplets from a single fertilized egg each year when they reproduce, thus generating an apparently high reproductive rate (at least relative to other species of armadillos; see reviews in [16]-[18]).

In this paper we use 15 years of capture-mark-recapture (CMR) data from a population of armadillos in northern Florida in an attempt to explore various demographic parameters that might contribute to range expansion. Specifically, we build on a previous study that focused on estimating apparent annual survival rate and transition probabilities between reproductive and non-reproductive states to estimate population growth rates. We then performed prospective perturbation analyses to quantify the relative influence of various demographic parameters on these estimates. A potential concern with these analyses was how our estimates might have been impacted by a three-year program of hardwood removal that eliminated much preferred habitat for armadillos at our study site [19]. Consequently, we used life-table response experiment (LTRE) analyses to decompose decreases in population growth rate due to logging into contributions from various demographic variables. Our findings represent the first rigorous analysis of population dynamics in nine-banded armadillos, and, thus, also provide the first formal attempt at identifying potential demographic mechanisms that might underlay the ongoing range expansion occurring in the United States. More broadly, our analyses provide data relevant to several theoretical issues in the study of biological invasions.

## Materials and Methods

### Ethics Statement

Permission to conduct fieldwork was provided by the Director of Research, Tall Timbers Research Station. All field procedures followed American Society of Mammalogists guidelines [20] and were approved by the animal care and use committee at Valdosta State University.

### Field Methods

Details of the field site and sampling methods can be found in [18], [19]. Briefly, data were collected at the Tall Timbers Research Station, located just north of Tallahassee, Florida during the summers (May-August) of 1992–2003. Within each year, we attempted to capture and mark, or in the case of previously marked individuals, identify all animals discovered during nightly censuses. Armadillos were captured using long dip nets. Once caught, individuals were weighed, sexed, measured, marked for temporary visual identification with various shapes and colors of reflective tape glued to different areas of the carapace, and marked for permanent identification by injection of a passive induced transponder (PIT) tag under the front carapace at its juncture with the neck.

Body mass was used to assign captured animals to one of three age categories: juveniles (young of the year) were individuals weighing <2 kg, yearlings weighed 2–3 kg, and adults weighed >3 kg [21]. Reproductive status of adult females was determined from inspection of the nipples as (1) definitely lactating, (2) possibly lactating, or (3) definitely not lactating [21]. We treated the first two categories as representing the reproductively active females in the population each year, however, because all adult males are physiologically capable of reproduction [22] we were unable to distinguish between reproductive and non-reproductive individuals (see [19]).

Although our fieldwork ended in 2003, some data were available from 2004–2006 because of the harvesting of armadillos at Tall Timbers in order to remove nest predators of northern bobwhite (*Colinus virginianus*; see [23]). We were granted access to these specimens in order to identify any individuals that had been captured and marked as part of our earlier sampling, and data from those animals are included here.

### Matrix Population Model and Deterministic Demographic Analysis

We constructed and analyzed stage-structured matrix population models, focusing on the female segment of the population because, as mentioned above, it was not possible to obtain reliable estimates of reproductive parameters for males.

We considered 4 stages, based on age and reproductive status: <1 year old = juveniles; ≥1 and <2 year old = yearlings; and ≥2 years old = adults [19]. Juveniles survive with annual survival probability *S_j_* and all survivors become yearlings the following year. Yearlings survive with annual survival rate *S_y_* and all surviving yearlings become non-reproductive adults the following year. Non-reproductive and reproductive adults survive the year with annual survival rate *S_n_* and *S_r_*, respectively. Additionally, non-reproductive adult females that survive the year become reproductive adults the following year with probability ψ*_nr_*, and remain non-reproductive with probability (1 - ψ*_nr_*). Finally, reproductive adult females that survive the year become non-reproductive adults the following year with probability ψ*_rn_*, and remain reproductive with probability (1 - ψ*_rn_*). The stage-structured population projection matrix was of the form:
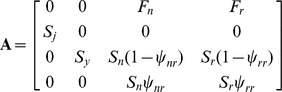
where *F_n_* and *F_r_* are fertility rates for non-reproductive and reproductive adults. Fertility rates were estimated using post-breeding census methods [24] as:

*F_n_* = 0.5 * *LS* *γ * *S_n_* * ψ*_nr_* and *F_r_* = 0.5 * *LS* *γ * *S_r_* * ψ*_rr_*, where *LS* is litter size and γ is a composite parameter that quantifies the probability of survival until trappable age.

All parameters except *LS* and γ were estimated using a multistate capture-mark-recapture (CMR) modeling framework [19]. Although the most parsimonious model [19] did not include a sex effect on survival, an equally well supported model (ΔAIC = 1.55) included an additive effect of sex and reproductive states on survival probabilities. Because our population model was limited to females only, and survival estimates obtained from the two models were very similar, we used this latter model to obtain estimates of survival and transition probabilities (and their variances and covariances) for females (see Figure S1).

We did not have reliable, field-based estimates of reproductive parameters. However, all available evidence indicates females give birth just once per year, and invariably produce litters of genetically identical quadruplets from a single fertilized egg (via obligate polyembryony, see [18], [25]). Thus, we assumed that *LS* was 4. Next, we created a variable, γ, to represent the proportion of quadruplets that survive to trappable age. Trappable age begins at first emergence of juveniles from their natal burrows (at ∼ 6–7 weeks old [18]). Note that this is a minimum time interval; time to actual capture can vary considerably beyond the date of first emergence. Multiple lines of evidence indicate that survivorship of all four littermates is low (review in [18]). Thus, it seems unlikely that γ would typically approach 1.0. However, we did not have sufficient data to identify a specific, well-supported point estimate of γ. Consequently, rather than limit our analyses to a single, arbitrarily picked value, we repeated them for a series of values ranging from 0 to 1.0.

Using the population projection matrix thus parameterized, we followed Caswell [24] to estimate deterministic finite population growth rate (λ), stable stage distribution, reproductive values, and elasticity of λ to changes in entries of the population projection matrix, as well as lower-level vital rates. The delta method was used to estimate variance and confidence intervals of λ [24]. For this, we obtained a variance-covariance matrix for stage-specific survival and transition probabilities directly from the CMR analysis [19]. Estimates of variances for *LS* and γ were not available, and so were assumed to be zero.

During the course of the study an extensive hardwood removal was conducted that eliminated much of the habitat favored by armadillos [26]. Previous work showed that state-specific survival rates of all animals were lower during the logging period than before or after [19]. Thus, in addition to estimating λ across all years of the study as a whole, we also performed demographic analyses separately for the years before (1992–1997), during (1998–2000), and after (2001–2006) hardwood removal.

### Life-table Response Experiment (LTRE) Analysis

To further examine the impact of hardwood removal on population dynamics, we used a fixed effect LTRE analysis [24], [27], [28] to decompose any change in λ due to hardwood removal into contributions from various vital rates, primarily, stage-specific survival. We expected lower population growth rate during and after hardwood removal than in the years prior to removal. Consequently, we used vital rates and λ prior to hardwood removal as a reference, and decomposed the difference in λ (Δλ) between the reference and treatments (during or after hardwood removal) as:
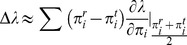

[24], [29], [30]; *π_i_* is a lower-level vital rate, and superscripts *r* and *t* refer to reference (before hardwood removal) and treatment (during or after hardwood removal). The term 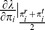 indicates that sensitivities were evaluated at the mean values of *π_i_*.

### Stochastic Demographic Analysis

Deterministic demographic analyses assume that the environment is constant, and there is no variability in vital demographic parameters. In reality, however, the environment as well as vital rates can vary unpredictably. Stochastic demographic methods allow explicit consideration of variability in vital rates. We used a simulation-based approach (50,000 steps) to estimate stochastic population growth rate and stochastic elasticities [24], [31]. We assumed that demographic parameters estimated using data collected before, during and after hardwood removal represented good, poor and moderate environmental conditions for our study population. We further assumed that these three environmental states were independently distributed with observed probabilities 0.4, 0.2, and 0.4, respectively. The stochastic population growth rate, , was calculated as: 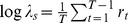 where *r_t_* = log(*n*(*t*+1)/*n*(*t*)) is a one-step population growth rate, *n*(*t*) and *n*(*t*+1) are projected population sizes at time *t* and *t*+1, respectively, and *T* = 50,000 steps [24], [31]. Variance of 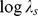 was estimated using log-normal approximation [24]. Elasticity of 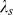 to matrix entries was calculated as:

where **u**(t) and **v**(t) are stochastic stage structure and reproductive value vectors at time *t*, *λ(t)* is 1-time step population growth rate, and the term 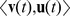 is the scalar product of vectors **v**(t) and **u**(t). Following [31], [32], we calculated three types of stochastic elasticities: (1) overall stochastic elasticities 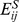 were calculated by setting 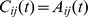 for every year *t*; (2) elasticities of *λ_s_* to the mean of matrix elements 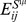 were obtained by setting 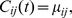 and (3) elasticities of *λ_s_* to the variance of the matrix entries 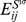 were obtained by setting 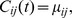 and 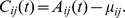 Elasticities of *λ_s_* to lower-level vital rates were calculated using methods described by Caswell [33].

All analyses were performed using programs written in MATLAB (Mathworks, Inc., Natick, MA).

## Results

### Population Dynamics across All Years

**Figure 1:**
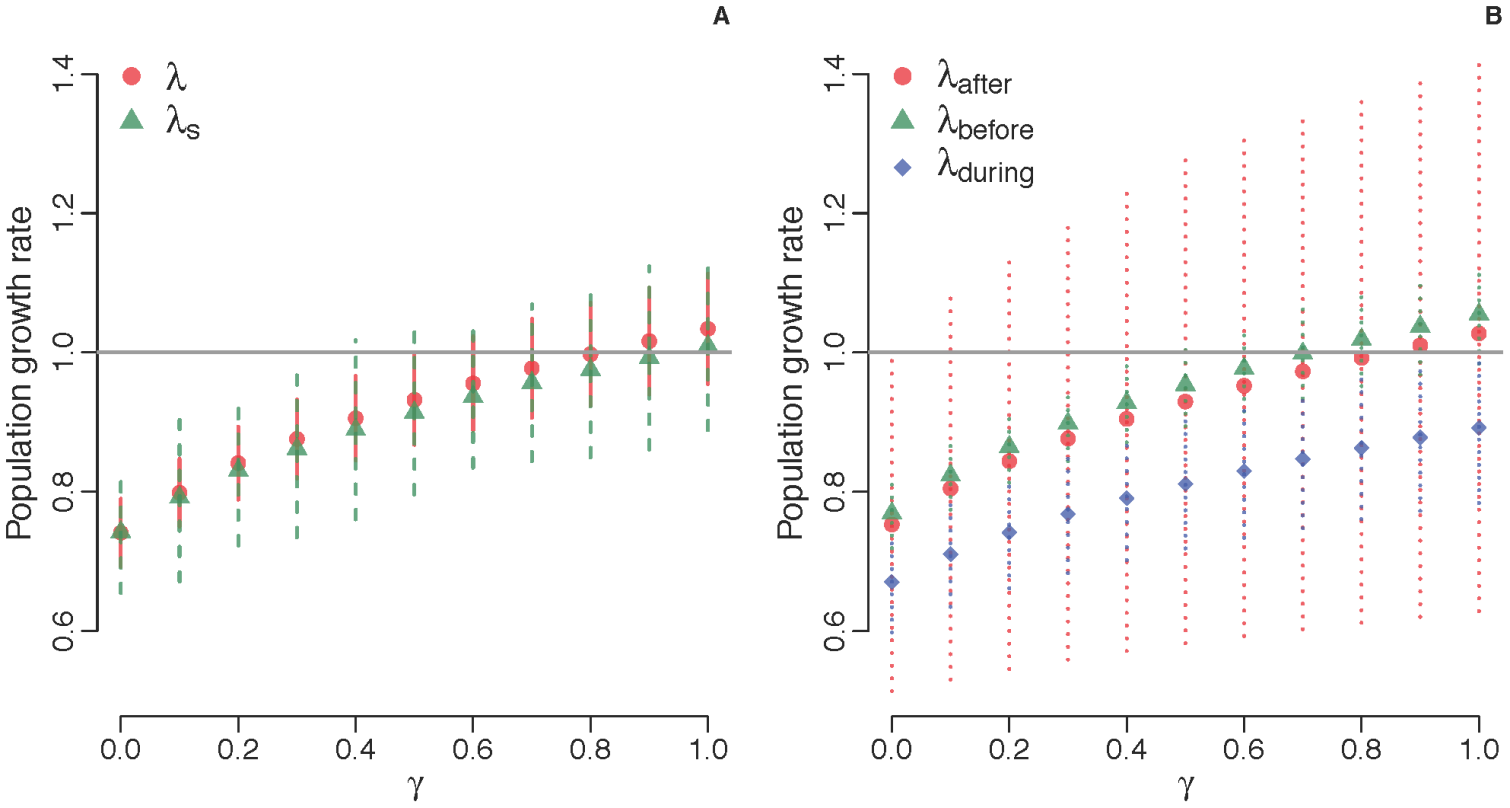
Annual population growth rate estimates as a function of γ. Gamma is the probability of a juvenile surviving to trappable age. (A) Estimates of deterministic (λ) and stochastic (λ_s_) growth rate across all years of the study. (B) Estimates of deterministic growth rate before (λ_before_), during (λ_during_), and after (λ_after_) hardwood removal. Vertical lines represent ±1 SE. There was considerable overlap in estimates provided by deterministic and stochastic projection models.

**Figure 2:**
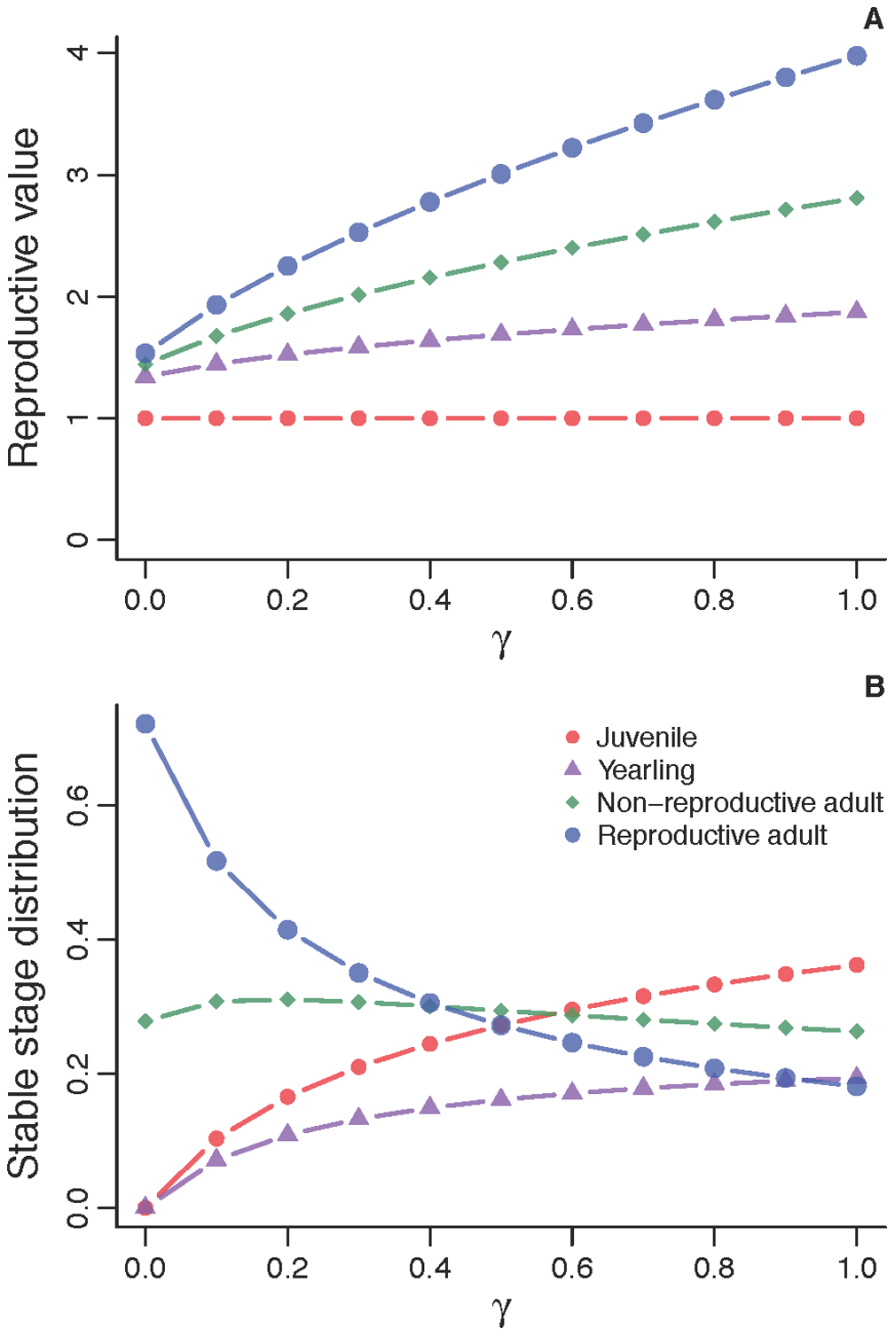
Stage-specific reproductive value (A) and stable stage distribution (B) as a function of γ. Gamma is the probability of a juvenile surviving to trappable age.

Overall estimates of demographic variables for the entire study period are presented in the Supplementary Materials (Figure S1). Across all years of the study, estimates of λ increased from 0.80–1.03, depending on the value of γ (Figure 1). Growth rates ≥1.0 were attained only with values of γ ≥0.80; the upper limits of 95% confidence intervals for λ were <1.0 for γ ≤0.50. Likewise, for γ ≤0.85, net reproductive rates were <1 (Figure S2), suggesting that most females did not replace themselves, except in unlikely scenarios where an average of ≥3.5 of the quadruplets survived to trappable age. Reproductive values and stable stage distributions also varied with γ, with an increase in reproductive value of adult stages as γ increased (Figure 2), and, as expected, a higher proportion of juveniles in the population with increased values of γ (Figure 2). Estimates of life expectancy indicated that juvenile armadillos were expected to live for 2.98±2.99 (SE) years.

**Figure 3:**
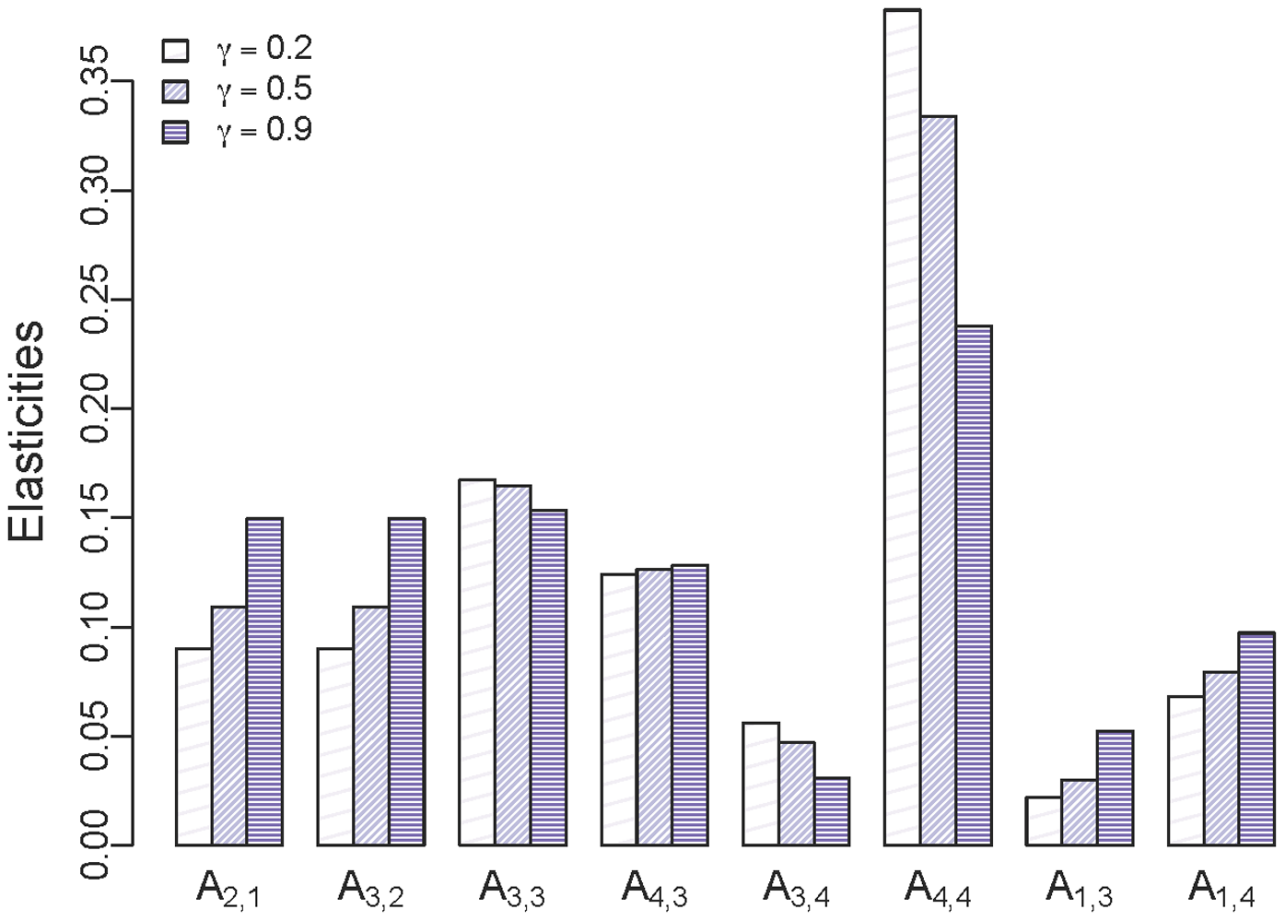
Elasticity of annual deterministic population growth rate (λ). Elasticities are presented as entries of the population projection matrix for three values of γ. X-axis labels (i.e., matrix entries) are: **A**_2,1_ = survival of juveniles; **A**_3,2_ = survival of yearlings; **A**_3,3_ = probability of surviving and remaining in the non-reproductive adult stage; **A**_4,3_ = probability of surviving and transitioning to the reproductive adult stage; **A**_3,4_ = probability of surviving and transitioning from the reproductive adult stage to the non-reproductive adult stage; **A**_4,4_ = probability of surviving and remaining in the reproductive adult stage**; A**_1,3_ = fertility rate of non-reproductive adults; and **A**_1,4_ = fertility rate of reproductive adults.

Matrix entry elasticities revealed that λ was proportionately most sensitive to changes in the probability of surviving and remaining in the reproductive adult stage, followed by the probability of surviving and remaining in the non-reproductive adult stage (Figure 3). As the value of γ increased, elasticity of λ to probability of surviving and remaining in the reproductive adult stage decreased with a corresponding increase in elasticity of λ to other entries of the population projection matrix (Figure 3). The reproductive adult stage was generally the most influential life stage except when γ ≈ 1 (Figure 3).

**Figure 4:**
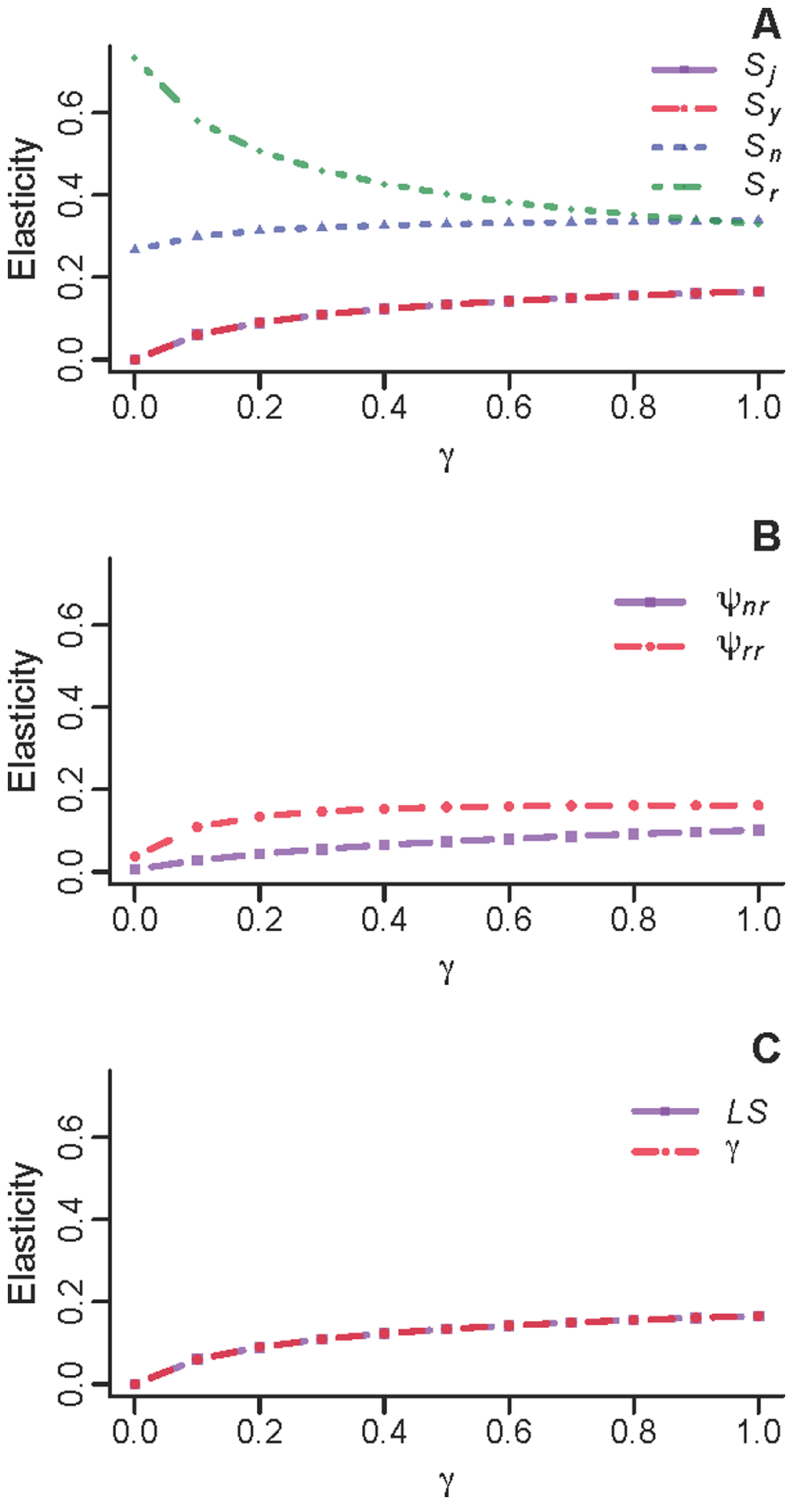
Elasticity of annual deterministic population growth rate (λ)to vital demographic parameters. Elasticities are presented for (A) survival, (B) reproductive transitions, and (C) litter size and gamma. Symbols are: *S_j_*, *S_y_*, *S_n_*, and *S_r_* = survival of juveniles, yearlings, non-reproductive adults and reproductive adults, respectively; ψ*_nr_* = probability of transitioning from non-reproductive to reproductive adult stage; ψ*_rr_* = probability of reproductive adults remaining reproductive adults; *LS* = litter size; and γ = probability of surviving to trappable age.

Elasticity of λ to lower-level vital rates identified *S_r_*, followed by *S_n_* and ψ*_rr_*, as the most influential vital rates across all reasonable values of γ (Figure 4). As the value of γ increased, elasticity of λ to *S_r_* decreased, with a corresponding increase in elasticity of λ to other vital rates; elasticity to *S_n_* slightly exceeded that to *S_r_* when γ ≈ 1 (Figure 4). The relative importance of reproductive parameters and survival of younger age classes was generally low, but increased as γ increased (Figure 4).

### Effects of Logging

Estimates of population growth rate were highest before, and lowest during, hardwood removal for all values of γ (Figure 1). Before hardwood removal, λ approached 1 for γ ≈ 0.75; λ never approached 1.0 during or after the hardwood removal, even when γ ≈ 1 (Figure 1). However, estimates were less precise for the logging and post-logging time frames (Figure 1), probably because of small sample sizes. Patterns of elasticities were similar to those described previously for the overall population (results not shown).

**Figure 5:**
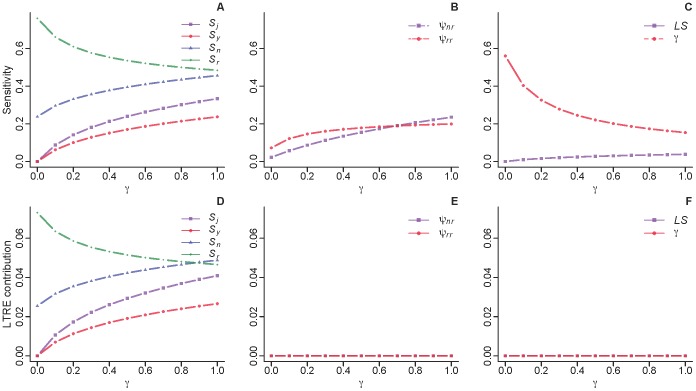
Results of the life table response experiment (LTRE) analysis. Deterministic population growth rates (λ) were compared before versus during hardwood removal. (A-C) Sensitivity of λ to vital rates. (D-F) LTRE contributions of vital rates to observed differences in λ. Note that four vital rates (i.e., transition rates for non-reproductive and reproductive adults, litter size, and γ) did not contribute to observed differences in population growth rate because separate estimates of these vital rates before and during hardwood removal were unavailable.

LTRE analysis revealed that the difference in survival of reproductive adults, followed by that of non-reproductive adults, contributed the most to observed differences in λ. However, the contribution of survival of reproductive adults decreased, and that of non-reproductive adults and juveniles increased, as the value of γ increased; these three vital rates contributed almost equally when γ ≈ 1 (Figure 5). This change in the pattern of vital rate contribution to λ was due primarily to an increase in the sensitivity of λ to the latter two variables (and a corresponding decrease in that to reproductive adults). Results of LTRE analysis comparing demography before and after hardwood removal were generally similar to those described above (results not shown).

### Stochastic Analyses

Stochastic population growth rates (λ_s_) were slightly lower than deterministic ones, but exhibited a similar relationship with γ (Figure 1). Patterns of elasticity of λ_s_ to mean vital rates and overall stochastic elasticities were similar to those of deterministic elasticities (Figure S3). However, elasticities of λ_s_ to all standard deviations of vital rates were negative, indicating that increases in variances of these rates reduced λ_s_ (Figure S3). Interestingly, λ_s_ was proportionately most sensitive to both the mean and standard deviation of survival of reproductive adults, followed by that to the mean and standard deviation of survival of non-reproductive adults for most values of γ (Figure S3).

## Discussion

The remarkable success of nine-banded armadillos in colonizing much of the southern United States has been puzzling because studies of reproductive success [34] and juvenile mortality [35] seem to indicate low recruitment [36]. The analyses reported here reinforce that view. Indeed, estimates of λ were <1.0 for values of γ ≤0.80 (the probability of a juvenile surviving to trappable age). Field observations suggest that high values of γ are unlikely. For example, data from three sites (including Tall Timbers) each showed that the modal litter size of captured juveniles was one, and that, across all sites, 468 juveniles from 283 litters were captured [18]. Assuming a fixed litter size of four, this means 664 (58.7%) juveniles were not caught. Whether these missing individuals died, dispersed, or remained in the population and somehow evaded capture is unknown, but to the extent these data indicate potentially low values of γ in populations of armadillos, it seems reasonable to conclude that range expansion has been achieved despite low population growth rates.

Such an assertion may be misleading for two reasons. First, irrespective of the species involved, successful biological invasions generally proceed in a more or less predictable sequence [37], [38]. Invasion begins with the establishment phase during which the invasive species colonizes a novel habitat and establishes itself. Once well established, and population density exceeds the Allee threshold, populations exhibit unregulated exponential growth, leading to the expansion phase. During expansion, dispersing propagules spread out from the initial site of invasion, creating an invasion front where the population may continue to grow exponentially. Finally, the spread of invasion may slow down or stop due to environmental constraints or other regulatory mechanisms during the saturation phase [37]. As the invasion front moves forward, population growth may slow down or cease altogether at the interior of the habitat due to density-dependent population regulation [39]. Indeed, theoretical models of invasion dynamics assume density-dependent population dynamics, whereby population growth depends on location relative to the invasion front, and local population density [40]-[43].

Nine-banded armadillos were first recorded in the Tallahassee area in 1972 [44]. Thus, the population at Tall Timbers was likely in place for about 20 years by the time we began our sampling, and was no longer at the leading edge of range expansion. Consequently, an alternative interpretation of our results might be that features of the Tall Timbers population do not reflect conditions occurring along the invasion front, but are instead more representative of a population in the saturation phase. Although such a proposal is consistent with theoretical expectations regarding biological invasions [37]-[43], unfortunately, no data are currently available to test this hypothesis because no populations along the northern limit of the species’ distribution have been studied. Nonetheless, assuming such a scenario is valid, our data suggest that populations of armadillos may become established quickly, with periods of high population growth being quite brief.

A second consideration is that our estimates of stage-specific survival rates were apparent survival rates. As such, these estimates cannot distinguish between death and dispersal. Given that range expansion is ongoing, dispersal undoubtedly occurs; failure to account for dispersal would lead to underestimation of population growth rate [30]. It is therefore likely that we have underestimated λ by confounding death and dispersal. Thus, although superficial examination of our results might suggest our population was in decline, and perhaps on the way to local extinction, the population may in fact be stable, as predicted by theoretical models of invasion dynamics [40], [41]. Some support for this position comes from the fact that even though armadillos were systematically culled from Tall Timbers during 2004–2006, the number of armadillos collected each year during this period was remarkably stable [23]. The most likely explanation for this was that large numbers of individuals were available in surrounding areas that immigrated and swiftly replaced removed residents. This would imply that dispersal rates can be quite high, which could in turn lead to maintenance of a relatively stable population, despite apparently low growth rates.

The models of population dynamics developed here were only moderately helpful in explaining the range expansion of nine-banded armadillos in the United States, but they are consistent with recent theoretical expectations regarding biological invasions. For example, as discussed above, because our population was no longer at the leading edge of expansion, we did not find evidence of high population growth rates, just as theory predicts [37], [38], [40], [41]. Likewise, recent theory has deemphasized the importance of reproductive parameters in determining invasive success, instead focusing on life-history strategies that favor investment in future, as opposed to current, reproduction [4]. Our data support this hypothesis. Both matrix entry and lower-level elasticity analyses indicated that survival parameters were generally more influential than reproductive parameters, and that adult stages (both reproductive and non-reproductive) were the most valuable in terms of relative contribution to population growth. Thus, for armadillos, the success of any particular reproductive event may be less critical than the capacity to survive and reproduce again.

Our life expectancy estimate of 3 years might argue against the importance of survival and future reproduction as important components of population growth in armadillos. Loughry and McDonough [18] reported that captive animals may live >20 years, and that ages of some animals at Tall Timbers exceeded ≥10 years. Based on this, they estimated that longevity in the wild might be about 8–12 years. Nonetheless, they also reported that the average (± SD) number of years juveniles recruited into the Tall Timbers population remained there was 3.83±2.34 years, and that the average tenure of animals first caught as adults and retained in the population was 2.89±2.06 years. These data suggest we have not severely underestimated life expectancy. Also, the large variance around our life expectancy estimate suggests some animals may be relatively long-lived, as indicated by the field data. In any case, a challenge for future work will be to determine the details of how survival and future reproduction influence the population dynamics of armadillos.

As argued above, it is debatable whether the Tall Timbers population was in general decline or not. Nevertheless, we did find that hardwood removal was associated with a substantially lower population growth rate (see also [19], [26]). A number of studies have identified bottomland hardwoods as preferred habitat for armadillos (review in [18]), and our analyses reinforce the view that eliminating such areas has serious negative consequences, probably by promoting increased emigration from logged sites. Because hardwood removal was followed by three years of culling armadillos as part of the predator removal experiment, we were unable to fully evaluate the long-term consequences of logging on our population. Even so, the fact that population growth rates were highest prior to logging, lowest during removal, and intermediate after the completion of logging suggests that hardwood removal not only directly impacted the population during the logging period, but that negative effects continued to persist in the population for an extended time thereafter. Thus, hardwood removal may represent one form of human disturbance that nine-banded armadillos do not tolerate well. Not surprisingly, the same may be true for many other species of armadillos also [7].

Fundamentally, for range expansion to occur some individuals must leave the place where they were born to colonize new areas. Populations of armadillos seem to consist of a core of long-term residents that move very little over time, and about an equal number of transients that are caught a few times as they move through an area but are rarely seen again (review in [18]). Presumably, it is transients that contribute the most to range expansion. Unfortunately, what determines whether an individual becomes a resident or a transient is unknown. Thus, even at the individual level, many of the factors promoting range expansion in nine-banded armadillos remain mysterious. An interesting project for the future would be to integrate population-level analyses of the type reported here with information on behavioral phenotypes (resident versus transient) to investigate whether the proportions of residents and transients are affected by changes in population dynamics. Perhaps such an approach will provide the insights necessary to better understand how armadillos have achieved such impressive success in colonizing the United States.

## Supporting Information

Figure S1 **Estimates of vital demographic parameters.** Symbols are: *S_j_*, *S_y_*, *S_n_*, and *S_r_* = survival of juveniles, yearlings, non-reproductive adults and reproductive adults, respectively; ψ*_nr_* = probability of transitioning from non-reproductive to reproductive adult stage; ψ*_rr_* = probability of reproductive adults remaining reproductive adults. Bars represent ±1 SE.
(TIFF)

Figure S2 **Net reproductive rate as a function of γ.** Net reproductive rate approaches 1.0 when γ ≈ 0.8.
(TIFF)

Figure S3 **Elasticity of stochastic population growth rate (λ_s_).** Elasticities are presented for (A) mean, and (B) standard deviation (SD) of vital demographic parameters for a range of values of γ. Symbols are: *S_j_*, *S_y_*, *S_n_*, and *S_r_* = survival of juveniles, yearlings, non-reproductive adults and reproductive adults, respectively; ψ*_nr_* = probability of transitioning from non-reproductive to reproductive adult stage; ψ*_rr_* = probability of reproductive adults remaining reproductive adults; *LS* = litter size; and γ = probability of surviving to trappable age.
(TIFF)
