# Supplementary figures and images for: Population Dynamics and Range Expansion in Nine-Banded Armadillos
Source: PLoS One. 2013 Jul 3;8(7):e68311. doi: 10.1371/journal.pone.0068311 (PMC3700932; doi:10.1371/journal.pone.0068311)

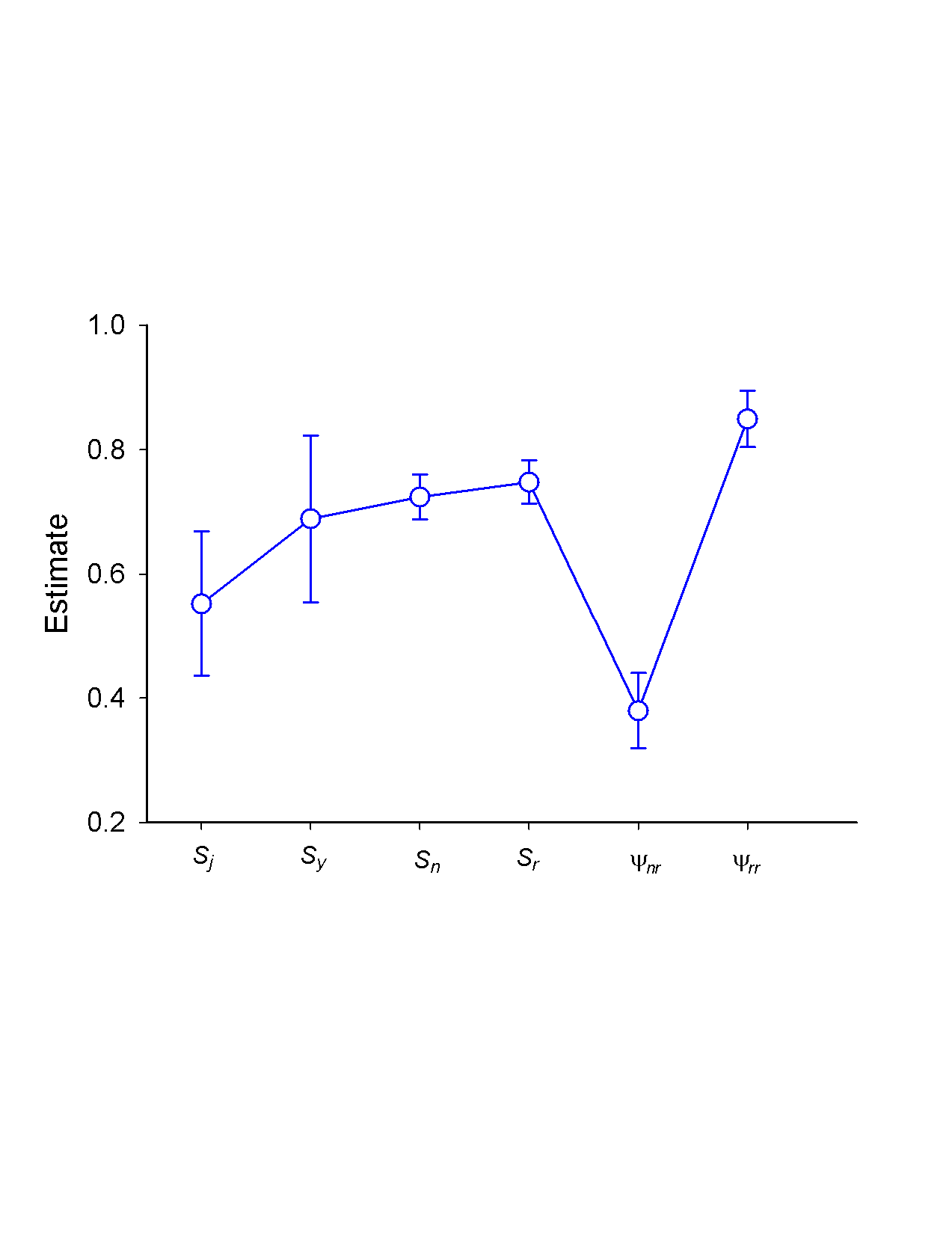

Supplement: Figure S1 — Estimates of vital demographic parameters. Symbols are: Sj, Sy, Sn, and Sr = survival of juveniles, yearlings, non-reproductive adults and reproductive adults, respectively; ψnr = probability of transitioning from non-reproductive to reproductive adult stage; ψrr = probability of reproductive adults remaining reproductive adults. Bars represent ±1 SE. (TIFF) [file pone.0068311.s001.tiff]

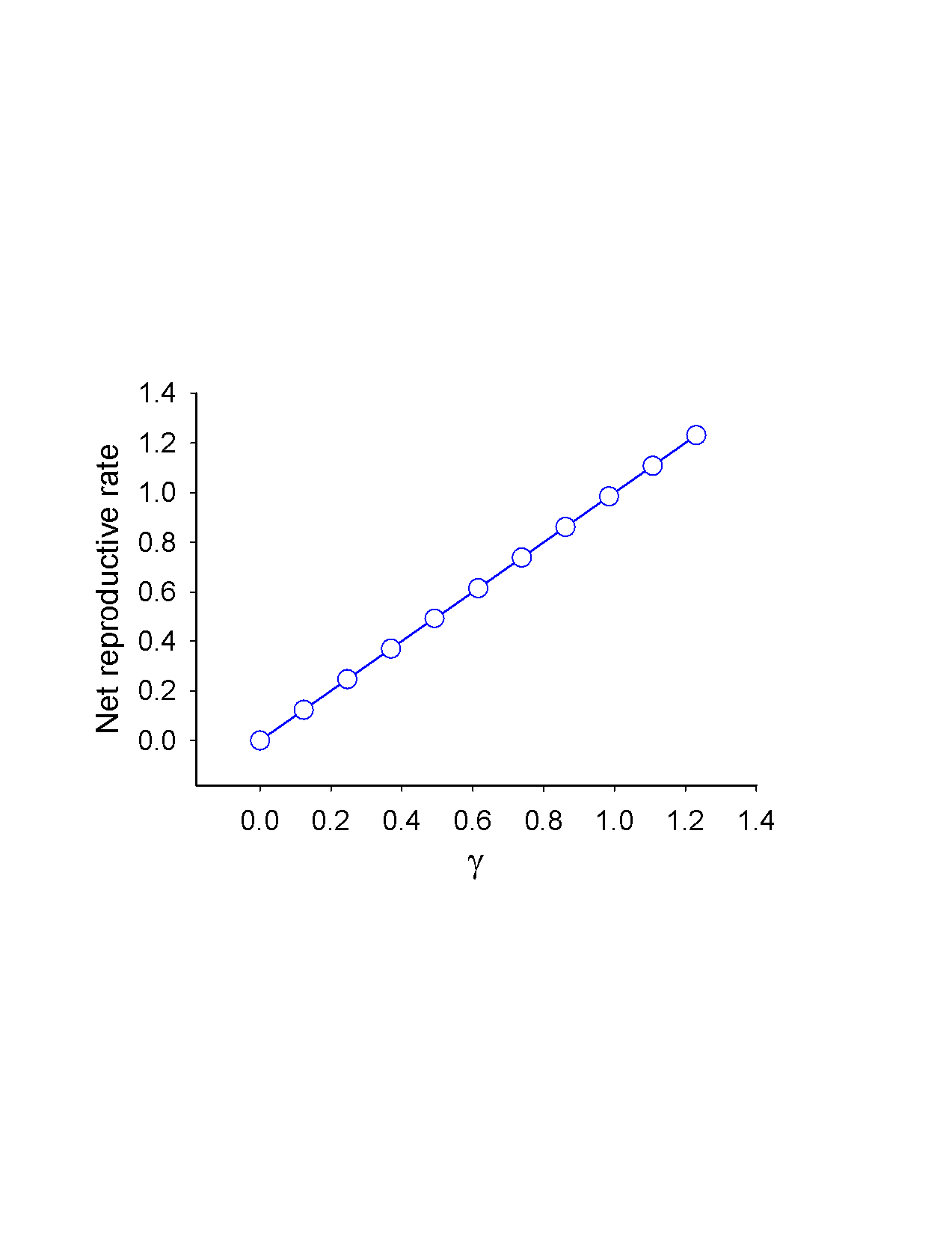

Supplement: Figure S2 — Net reproductive rate as a function of γ. Net reproductive rate approaches 1.0 when γ ≈ 0.8. (TIFF) [file pone.0068311.s002.tiff]

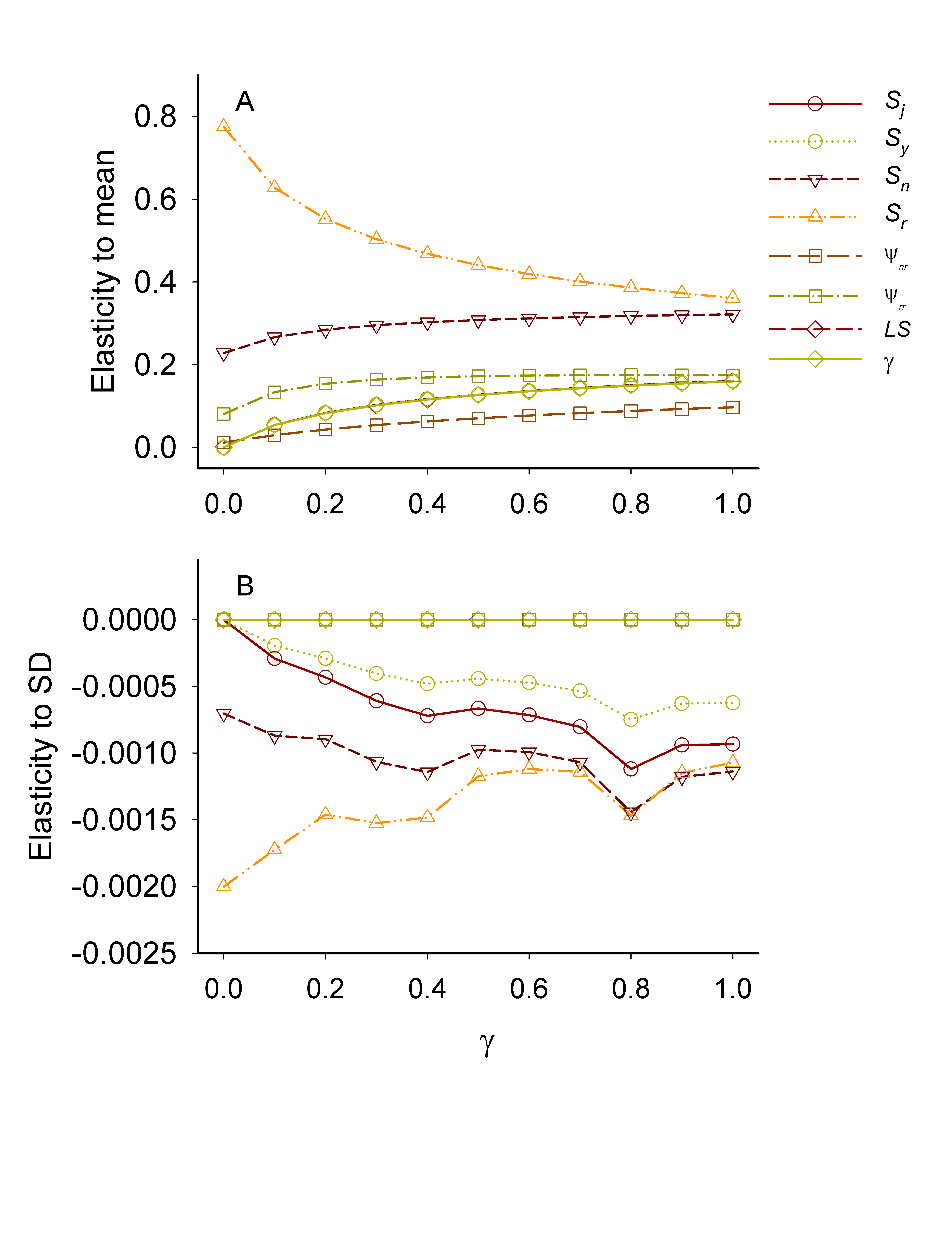

Supplement: Figure S3 — Elasticity of stochastic population growth rate (λs). Elasticities are presented for (A) mean, and (B) standard deviation (SD) of vital demographic parameters for a range of values of γ. Symbols are: Sj, Sy, Sn, and Sr = survival of juveniles, yearlings, non-reproductive adults and reproductive adults, respectively; ψnr = probability of transitioning from non-reproductive to reproductive adult stage; ψrr = probability of reproductive adults remaining reproductive adults; LS = litter size; and γ = probability of surviving to trappable age. (TIFF) [file pone.0068311.s003.tiff]
